# Case Report: Experience of a case of cardiac arrest caused by postoperative pulmonary embolism and hepatic rupture induced by cardiopulmonary resuscitation

**DOI:** 10.3389/fcvm.2025.1624064

**Published:** 2025-09-08

**Authors:** Haowen Yang, Yuehong Li, Xiongbing Peng, Zhaojun Li, Peiwan Liu, Xingbao Fang

**Affiliations:** ^1^ The Affiliated Qujing Hospital, Kunming Medical University, Qujing, Yunnan, China; ^2^ Department of Hepatobiliary and Pancreatic Surgery, The First People's Hospital of Qujing, Qujing, Yunnan, China

**Keywords:** case report, multidisciplinary treatment, liver rupture, cardiopulmonary resuscitation, pulmonary embolism

## Abstract

Postoperative pulmonary embolism (PE) poses a serious threat to a patient's life. However, cases involving concurrent liver rupture are exceedingly rare. This report describes a case of a patient who experienced cardiac arrest due to PE following lower extremity varicose vein surgery, followed by liver rupture during cardiopulmonary resuscitation (CPR). Under the timely intervention of a multidisciplinary team (MDT), the patient underwent extracorporeal membrane oxygenation (ECMO)-assisted resuscitation and hepatic repair surgery. Through a staged anticoagulation strategy, a balance was achieved between bleeding control and thrombosis prevention. Ultimately, after 23 days of treatment, the patient regained consciousness and was transferred to rehabilitation.

## 1 Introduction

Postoperative PE is a life-threatening complication, and its occurrence in combination with hepatic rupture is extremely rare. Retrospective studies have shown that autopsy data indicate a higher incidence of liver injury than clinical reports. In such cases, one of the greatest challenges in treatment lies in the inherent conflict between the need for systemic anticoagulation for PE and ECMO, and the need for hemostasis for liver rupture. How to better apply anticoagulant therapy has become a question. This case report describes the first instance of PE occurring after lower extremity varicose vein surgery, which led to liver rupture during CPR. It is hoped that this case will provide guidance for future management of such situations.

## 2 Case presentation

A 69-year-old female with a 7-year history of lower limb varicose veins presented with progressive dilation of superficial veins in the right lower limb, accompanied by occasional swelling and pain. Her height was 152 cm, weight 61 kg, and body surface area (BSA) was calculated as 1.61 m^2^. The Caprini score was 3, indicating moderate risk, while VTE risk assessment for bleeding showed low risk. Due to the patient's history of upper gastrointestinal bleeding, according to the 2023 American Society for Vascular Surgery (SVS) Clinical Practice Guidelines for Lower Extremity Varicose Veins, no pharmacological anticoagulant therapy was administered as part of deep vein thrombosis (DVT) prophylaxis prior to surgery.

### 2.1 Key findings

• Cardiac arrest due to massive postoperative pulmonary embolism following varicose vein surgery.
• Severe CPR-associated hepatic rupture (AAST grade IV) identified shortly after resuscitation.
• Successful ECPR with VA-ECMO support, followed by emergency hepatic repair.
• A staged anticoagulation strategy balancing bleeding control and thrombosis prevention, with subsequent percutaneous pulmonary artery thrombectomy.
• Favorable recovery with consciousness regained and transfer to rehabilitation by postoperative day 23.

### 2.2 Preoperative conditions

On February 24, 2024, the patient underwent high ligation and stripping of the great saphenous vein combined with sclerotherapy under regional anesthesia. Based on the preoperative assessment, postoperative DVT prevention included elastic compression, early mobilization, and ankle counterpulsation, without pharmacological anticoagulation.

On postoperative day 1, the patient experienced sudden syncope during mobilization, with bradycardia (40 bpm), hypotension (40/30 mmHg), and loss of consciousness. High-flow oxygen was delivered via face mask, and bedside CPR was immediately initiated. Epinephrine (1 mg × 9) and dopamine (20 mg × 2) were administered intravenously, followed by continuous infusions at 0.85 μg/kg/min and 10 μg/kg/min, respectively. Lactated Ringer's solution was also given. Emergency labs revealed a hemoglobin level of 104 g/L. Tracheal intubation was performed at minute 14, without the use of transesophageal echocardiography. Echocardiography showed right heart enlargement, and a central venous catheter was placed. CPR continued for a total of 39 min until ECMO support was established. During resuscitation, the patient intermittently regained consciousness and became agitated, but spontaneous circulation was not fully restored.

An MDT comprising ICU, anesthesiology, and neurology specialists was urgently assembled. After evaluation, the team decided to initiate extracorporeal cardiopulmonary resuscitation (ECPR). Under ultrasound guidance, a 17 Fr arterial cannula was inserted into the left femoral artery, along with the placement of a distal perfusion catheter to prevent limb ischemia. Subsequently, a 21 Fr venous cannula was inserted into the left femoral vein using the Seldinger technique. Intravenous heparin was administered at a dose of 40 U/kg. At 39 min after CPR initiation, veno-arterial ECMO was established, and stable extracorporeal circulation was achieved.

The initial pump rate was approximately 2,280 r/min, with a blood flow rate of approximately 2.2 L/min, SVO₂: 89%, Hct: 18%, and Sao₂: 99%. After ECMO was established, her pulse was 70 bpm and her blood pressure was 70/60 mmHg. Central venous catheterization indicated hypovolemia. Therefore, leukocyte-depleted red blood cells, fresh frozen plasma, and cryoprecipitate were administered for volume resuscitation.

**Figure 1:**
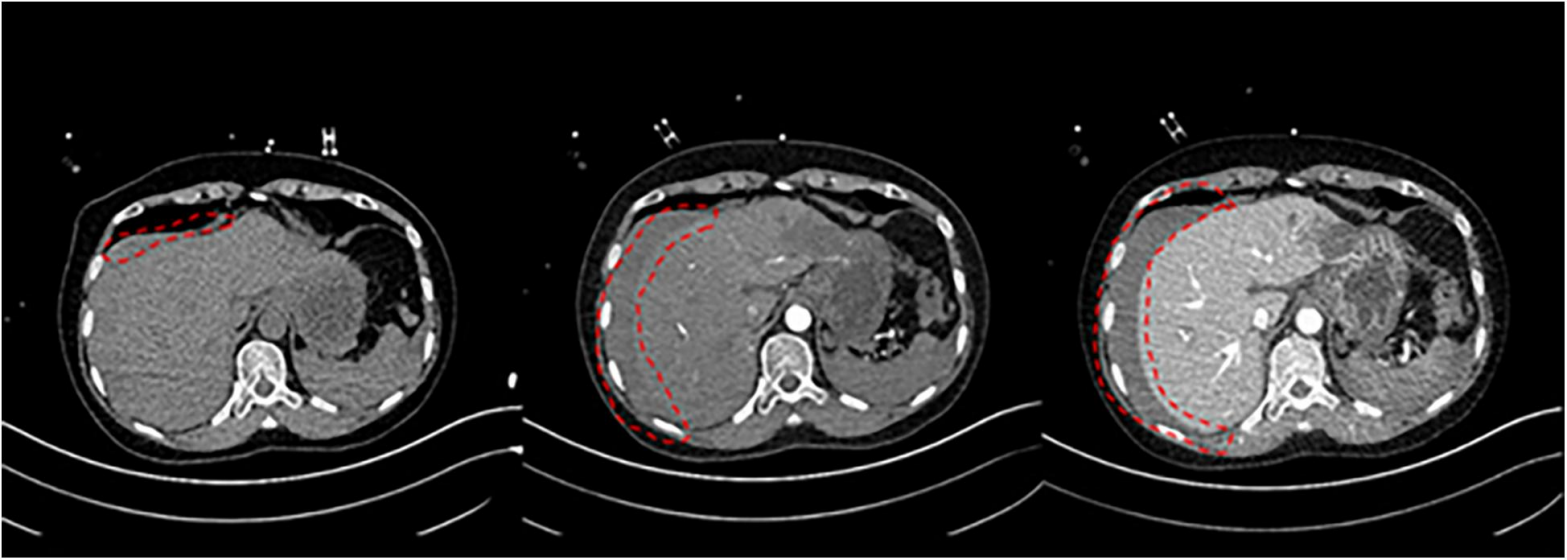
Non-contrast CT of the abdomen, arterial phase, venous phase.

**Figure 2:**
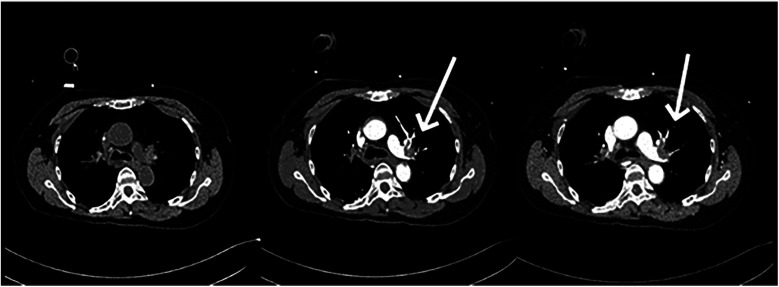
Non-contrast CTA of the pulmonary artery, arterial phase, venous phase.

11 min after ECMO was started, the patient was transferred to the CT room. Abdominal CT and pulmonary artery CTA revealed the following findings: (1) Bilateral pulmonary artery embolism. (2) Exudation in the lower lobes of both lungs and multiple rib fractures on both sides. (3) Abnormalities in the right posterior lobe and left lateral lobe of the liver (Figures 1, 2).

Subsequently, the patient was transferred back to the ICU for monitoring and treatment. At this time, multidisciplinary experts, including those from the Gastrointestinal Surgery Department and the Hepatobiliary Surgery Department, promptly convened a second MDT discussion. They highly suspected intra-abdominal hemorrhage and liver rupture. Therefore, 22 min after the CT scan, the patient was taken to the operating room for emergency exploratory laparotomy.

### 2.3 Surgical management

The patient underwent emergency liver repair under general anesthesia. Intraoperative exploration showed 2,000 ml of dark red blood in the abdominal cavity, aspirated along with 100 g of blood clots around the liver. The right hepatic lobe had active bleeding and a 12 × 10 cm hematoma. Classified as Grade IV by AAST and CT standards, the liver had ischemic damage, a fragile texture, and multiple lacerations, the largest 3 × 3 cm-with ongoing bleeding.

Bleeding vessels at the laceration were ligated, and gelatin sponges and hemostatic gauze were applied, with sutures to secure the edges. As the right posterior hepatic region had persistent oozing, the right hepatic artery was ligated, reducing bleeding significantly. Bleeding was controlled using absorbable gauze. Intraoperatively, the patient's hemoglobin was 91 g/L. Total blood loss was about 3,000 ml, and the patient received 12 units of leukocyte-depleted red blood cells, 20.5 units of cryoprecipitate, and 1,000 ml of frozen plasma during the operation. Post-surgery, the patient was transferred to the ICU due to a critical condition.

### 2.4 Postoperative course

**Figure 3:**
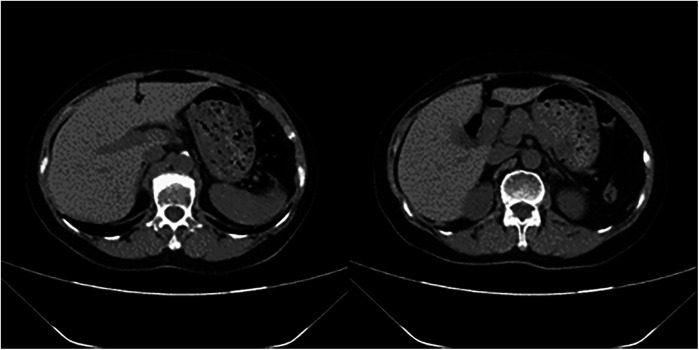
Abdominal CT before discharge.

At this point, the MDT intervened for the third time to establish a postoperative treatment plan. The patient underwent percutaneous pulmonary artery thrombectomy (INDIGO thrombectomy) combined with pulmonary angiography on the second postoperative day. On pulmonary arteriography, filling defects and thrombosis were seen in the left pulmonary artery trunk, left lower lung, right upper lung, and right lower lung. After repeated thrombus removal and reimaging, the filling defect of the right upper lung disappeared, the filling defect of the left main pulmonary artery and left lower pulmonary artery improved significantly. The end of the right lower lung was slightly poorly visualized; therefore, the procedure was concluded. Hemodynamic stability was achieved within 24 h post-thrombectomy. ECMO support was discontinued on the third postoperative day. Due to ongoing respiratory dependence, the patient underwent percutaneous tracheostomy on the 11th postoperative day. By the 23rd postoperative day, the patient had regained normal consciousness, shown clinical improvement, and been transferred to the rehabilitation department for continued rehabilitation therapy (Figure 3).

At the end of surgery, the patient's coagulation profile showed INR 1.46, APTT 47.2 s, and TT 29.9 s. To prevent rebleeding, systemic anticoagulation was withheld for 24 h. After improvement in coagulation parameters (INR 1.27, APTT 33.4 s, TT 18.8 s), a low-dose heparin infusion totaling 12,500 IU was administered solely to maintain ECMO cannula patency (i.e., to prevent line thrombosis). Systemic anticoagulation was resumed the following day after thrombectomy, using sodium citrate, heparin, argatroban, and rivaroxaban. Coagulation function was monitored every 8 h, and anticoagulant dosages were adjusted accordingly. During this period, INR ranged from 1.11 to 1.77, APTT from 31.3 to 75.4 s, and TT from 14.7 to 97.3 s. On postoperative day 15, anticoagulation therapy was simplified to oral rivaroxaban (10 mg/day), with gradual recovery of coagulation function.

The patient was comatose (GCS 3) upon admission to the ICU, with unequal pupil size and no light reflex. Subsequently, the GCS gradually improved, and the pupil reflex returned. Cranial CT showed lacunar cerebral infarction in the bilateral basal ganglia, with no acute hemorrhage. Video EEG showed no epileptic discharges or significant abnormalities. Upon discharge from the ICU, the patient was conscious and cooperative, with no focal neurological deficits, but had impaired muscle strength in all four limbs, requiring rehabilitation training.

## 3 Discussion

This case report describes an extremely rare and life-threatening surgical complication: a patient developed PE following lower extremity varicose vein surgery, which subsequently led to cardiac arrest. During CPR, the patient also experienced severe liver rupture. Although CPR-related injuries are well-recognized, clinically significant liver lacerations resulting from CPR remain extremely rare. Additionally, due to the urgent nature of the situation during resuscitation, such injuries are often overlooked.

Retrospective studies indicate that the clinically reported incidence of liver injury following CPR is significantly lower than the actual level revealed by autopsy findings. Clinical data indicate that the incidence of liver injury requiring surgical intervention is less than 1% (1), while autopsy data suggest that the actual rate may exceed 2% (2). This discrepancy suggests that a significant number of mild or asymptomatic liver injuries go unrecognized, potentially due to factors such as early hemodynamic instability during resuscitation, inadequate imaging diagnostic tools, or clinical prioritization of treating cardiovascular and pulmonary conditions.

The challenge in treating this case lies not in the isolated organ injury but in balancing the management of pulmonary artery embolism with controlling bleeding from liver rupture. The liver is the primary organ responsible for synthesizing clotting factors. Once ruptured, its coagulation function is significantly impaired. If systemic anticoagulation is initiated too early or at excessive doses, it may disrupt the liver's self-repair process and lead to more severe bleeding (3). Additionally, damaged liver tissue can enter the systemic circulation via the hepatic veins, forming secondary thrombi and exacerbating PE (4). Despite the risk of bleeding, pulmonary embolism and myocardial infarction are still the predominant causes of mortality. As such, evidence supports the continued use of anticoagulation and thrombolytic therapy during CPR to improve outcomes (5–7).

In this case, we adopted a phased, precision anticoagulation strategy based on the patient's condition and guidelines (ISTH, ELSO). During the early recovery phase, the MDT decided to temporarily withhold systemic anticoagulation to avoid uncontrolled bleeding. To maintain patent ECMO circulation, low-dose heparin (40 U/kg) was started at the 24th postoperative hour. Once a significant reduction in bloody drainage fluid was observed and coagulation parameters permitted, oral rivaroxaban and intravenous heparin infusion were resumed, with close monitoring of INR, TT and APTT. Anticoagulant use was adjusted daily based on real-time coagulation parameters (heparin: 14–20 U/kg-h, sodium citrate 35–80 g/day, rivaroxaban tablets, 5–10 mg/day) to achieve a dynamic balance between hemostasis and anticoagulation; the regimen was gradually transitioned to only oral anticoagulants to reduce the risk of heparin-induced thrombocytopenia (8). During ECMO support, the heparin pump rate was dynamically adjusted to promptly lower elevated APTT, ensuring anticoagulation efficacy while avoiding worsening of persistent intra-abdominal bleeding (9). This case highlights that a staged, individualized anticoagulation regimen is particularly critical for balancing hemostasis and anticoagulation in the management of complex multiple injuries complicated by acute PE. Grinberg et al. (10) also exemplify the concept that rapid recognition and aggressive surgical intervention is the key to breaking the vicious cycle of “bleed-anticoagulate-rebleed” when continuous intraoperative anticoagulation is unavoidable. Notably, our team performed percutaneous pulmonary artery thrombectomy when hemodynamically feasible. Percutaneous mechanical thrombectomy was performed to rapidly debulk thrombotic load. This created conditions for maintaining ECMO and liver recovery under low-dose, adjustable anticoagulation levels.

It should be noted that during the rescue operation, the MDT considered the possibility of intra-abdominal bleeding. However, due to the need for a comprehensive assessment of the patient's overall condition (head, PE, abdomen) and the influence of free blood in the abdominal cavity, the MDT decided to perform head and abdominal CT scans and pulmonary artery CTA examinations directly, rather than relying solely on ultrasound to locate the bleeding site. This strategy is also in line with Grinberg et al. (10), who recommended that systemic CT should be performed as early as possible in ECMO-supported patients with suspected hemorrhage, with the aim of identifying key complications before they worsen, thereby improving prognosis. In terms of surgical options, right hepatectomy was not feasible due to the patient's fragile liver and the risk of massive bleeding, ischemic injury, and disseminated intravascular coagulation (DIC). Therefore, a treatment plan combining liver repair and compression hemostasis was adopted.

**Figure 4:**
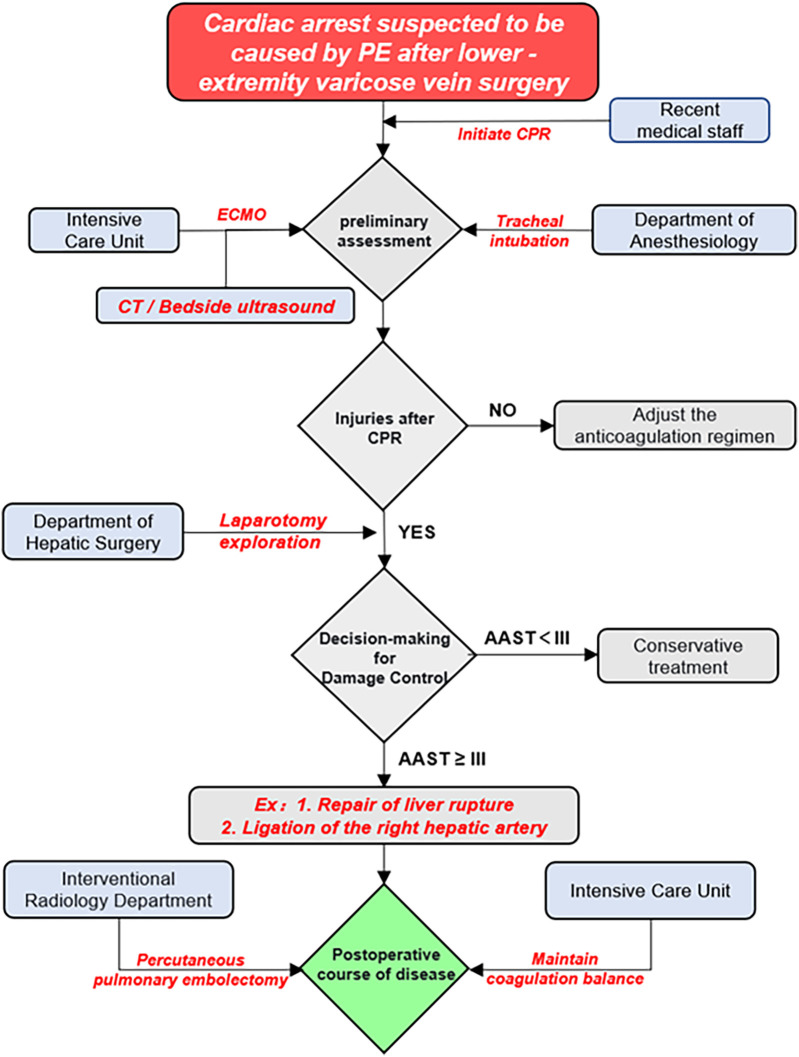
Process schematic diagram.

From a diagnostic and therapeutic standpoint, this case prompts reconsideration of the traditional post-CPR diagnostic workflow. FAST (Focused Assessment with Sonography for Trauma) or rapid CT should be used promptly to screen for possible intra-abdominal hemorrhage in patients who have received CPR for more than 10 min. For patients with coexisting PE and AAST Grade IV liver injury, prioritizing hemostasis—rather than conventional and thrombolysis-oriented pathways—appears critical (Figure 4).

## 4 Conclusion

We emphasize that high-level multidisciplinary collaboration is crucial for timely diagnosis and optimal treatment planning in such complex scenarios. A staged and individualized anticoagulation strategy helps clinicians balance the risk of thrombosis and bleeding. When feasible, timely mechanical thrombectomy can stabilize hemodynamics and improve clinical outcomes.

## Data Availability

The original contributions presented in the study are included in the article/Supplementary Material, further inquiries can be directed to the corresponding author.
